# Assessing Chitinases and Neurofilament Light Chain as Biomarkers for Adult-Onset Leukodystrophies

**DOI:** 10.3390/cimb46050262

**Published:** 2024-05-05

**Authors:** Paulo de Lima Serrano, Thaiane de Paulo Varollo Rodrigues, Leslyê Donato Pinto, Indiara Correia Pereira, Igor Braga Farias, Renan Brandão Rambaldi Cavalheiro, Patrícia Marques Mendes, Kaliny Oliveira Peixoto, João Paulo Barile, Daniel Delgado Seneor, Eduardo Gleitzmann Correa Silva, Acary Souza Bulle Oliveira, Wladimir Bocca Vieira de Rezende Pinto, Paulo Sgobbi

**Affiliations:** 1 PSEG Centro de Pesquisa Clínica, São Paulo 04038-002, SP, Brazil; paulo.serrano@psegtrials.com (P.d.L.S.); thaiane@psegtrials.com (T.d.P.V.R.); leslye@psegtrials.com (L.D.P.); indiara@psegtrials.com (I.C.P.); eduardo@psegtrials.com (E.G.C.S.); 2 Department of Neurology and Neurosurgery, Federal University of São Paulo (UNIFESP), São Paulo 04039-060, SP, Brazil; med_igor@hotmail.com (I.B.F.); renan.cavalheiro@psegtrials.com (R.B.R.C.); patriciamarquesmendes@gmail.com (P.M.M.); kalinyop355@gmail.com (K.O.P.); joao.barile@psegtrials.com (J.P.B.); danielseneor30@gmail.com (D.D.S.); acary.bulle@unifesp.br (A.S.B.O.); wladimirbvrpinto@gmail.com (W.B.V.d.R.P.)

**Keywords:** leukoencephalopathy, leukodystrophy, biomarker, chitotriosidase, CSF1R, adult-onset leukoencephalopathy with axonal spheroids and pigmented glia

## Abstract

Leukodystrophies represent a large and complex group of inherited disorders affecting the white matter of the central nervous system. Adult-onset leukoencephalopathy with axonal spheroids and pigmented glia (ALSP) is a rare leukodystrophy which still needs the proper identification of diagnostic, prognostic, and monitoring biomarkers. The aim of this study was to determine the diagnostic and prognostic value of chitinases and neurofilament light chain as biomarkers for ALSP. A cross-sectional study was performed to analyze cerebrospinal fluid levels of chitinases (chitotriosidase and chitinase 3-like 2) and neurofilament light chain in five different groups: (i) normal health individuals; (ii) patients with definitive diagnosis of ALSP and genetic confirmation; (iii) asymptomatic patients with CSF1R variants; (iv) patients with other adult-onset leukodystrophies; and (v) patients with amyotrophic lateral sclerosis (external control group). Chitinase levels showed a statistical correlation with clinical assessment parameters in ALSP patients. Chitinase levels were also distinct between ALSP and the other leukodystrophies. Significant differences were noted in the levels of chitinases and neurofilament light chain comparing symptomatic (ALSP) and asymptomatic individuals with CSF1R variants. This study is the first to establish chitinases as a potential biomarker for ALSP and confirms neurofilament light chain as a good biomarker for primary microgliopathies.

## 1. Introduction

The leukodystrophies represent a group of rare inherited disorders that primarily affect the white matter (WM) of the central nervous system (CNS). These disorders exhibit diverse genetic origins and substantial phenotypic variability, regardless of the structural white matter component involved, the underlying molecular process, and/or the disease course [1,2].

Leukodystrophies are characterized by primary pathology impacting glial cells and the myelin sheath, with a varied etiology that encompasses oligodendrocytes, astrocytes, ependymal cells, and microglia at the cellular level. The underlying pathological mechanisms show wide variation and may involve inborn errors of metabolism, disrupted protein biosynthesis, oxidative stress, and energy failure due to respiratory chain defects, as well as abnormalities in RNA metabolism such as abnormal mRNA translation and aberrant regulation of ribosomal RNA. Ultimately, these cellular and molecular processes will lead to abnormal findings in neuroimaging, particularly in magnetic resonance imaging (MRI) [1,2,3].

The occurrence of leukodystrophies is infrequent, with an estimated incidence ranging from 1 per 100,000 to 1 per 7600 cases. These disorders can be inherited through autosomal recessive or dominant, X-linked, or mitochondrial patterns, affecting individuals across diverse age groups and ethnicities [4,5,6].

From a radiological perspective, leukodystrophies can be broadly categorized into demyelinating and hypomyelinating types based on the appearance of WM signal on T1-weighted, T2-weighted, and fluid-attenuated inversion recovery (FLAIR) sequences in MRI. In hypomyelinating leukodystrophies, the WM exhibits diffuse and symmetric T2/FLAIR hyperintensity, and the T1 signal is hypointense (if hypomyelination is severe), isointense relative to the cortex, or hyperintense, depending on the amount of myelin deposited. In demyelinating types, the radiological presentation of WM is characterized by T2/FLAIR hyperintensity and T1 hypointensity, with variable topography and pattern [7,8].

Microglia are resident macrophages in the CNS exhibiting prominent and diverse functions during development and adulthood under both homeostatic and disease conditions. Remarkably, microglia self-renew through low-rate proliferation in combination with apoptosis throughout life, without any contribution from bone-marrow-derived monocytes to the microglial pool, at least under homeostatic conditions [9,10]. As the principal immune effectors within the CNS, they function as surveillance cells and sensors of pathological events. In the WM, microglia contribute to the regulation of myelin maintenance and play a crucial role in response to injury and during the repair process. Under normal conditions, microglia support the survival and differentiation of oligodendrocyte progenitor cells (OPCs) and facilitate myelination. In the event of injury, microglia exhibit dual roles, influenced by their polarization status. They may either impede OPC differentiation and induce oligodendrocyte apoptosis, or promote OPC differentiation and remyelination. Another significant aspect of microglial function involves their role in clearing myelin debris following WM damage and myelin loss. This clearance is a crucial step in the remyelination process, highlighting the importance of microglia in WM repair [9,10,11,12].

Microgliopathies refer to disorders resulting from mutations in genes predominantly expressed by microglia, leading either directly or indirectly to WM defects. Leukodystrophies associated with mutations in *CSF1R, TREM2*, and *TYROBP* genes can be classified as primary microgliopathies [3,13].

Adult-onset leukoencephalopathy with axonal spheroids and pigmented glia (ALSP) is a rare neurological disorder pathologically characterized by demyelination of cerebral WM, swollen axons, and pigmented glial cells. It is caused by autosomal dominant inherited variants in the *CSF1R* gene, leading to cognitive dysfunction and motor compromise. Cognitive dysfunction may manifest as cognitive impairment and various neuropsychiatric symptoms, including anxiety, depression, apathy, abulia, irritability, disinhibition, and distraction, either in isolation or progressing to the deterioration of higher cortical functions such as aphasia, agraphia, acalculia, and apraxia. The motor spectrum of the disease can clinically present as parkinsonism (with features like tremor, rigidity, bradykinesia, and postural instability), gait abnormalities due to spasticity or cerebellar ataxia, or bulbar signs such as slurred speech, dysphagia, and dysphonia. ALSP is a progressive disorder significantly impacting quality of life, culminating in the final stages with the loss of speech, involuntary movements, and severe cognitive impairment [14].

Current investigations are exploring the replacement of a depleted microglial niche, either through genetic methods or pharmacological interventions such as CSF1R inhibitors, or modulation of alternative targets like the triggering receptor expressed on myeloid cells 2 (TREM2) pathway for the treatment of microgliopathies and neurodegenerative disorders [14,15,16].

In this evolving scenario, the significance of identifying biomarkers capable of detecting disease progression and serving as valuable tools for monitoring therapeutic response or as outcomes in clinical studies is increasing. In this study, our aim was to determine the diagnostic and prognostic value of chitinases and neurofilament light chain as biomarkers for ALSP.

## 2. Materials and Methods

### 2.1. Patients and Individuals

A cross-sectional clinical study was conducted to collect and analyze levels of chitinases and NfL in patients diagnosed with ALSP. The participants were divided into five groups as follows: (A) healthy individuals with a negative genetic test for *CSF1R* mutations and no history of neurological disorders; (B) patients with a definitive diagnosis of ALSP with a confirmed mutation in the *CSF1R* gene; (C) individuals with a positive genetic test for a variant in the *CSF1R* gene who were asymptomatic and exhibited no evidence of clinical manifest ALSP; (D) patients with adult-onset leukodystrophy unrelated to the *CSF1R* gene; (E) patients with sporadic amyotrophic lateral sclerosis (sALS), serving as an external control group.

Every participant in group A underwent evaluation by two distinct neurologists (PS and PLS) who had expertise in the evaluation of neurodegenerative diseases. The purpose of this assessment was to validate the absence of any history or clinical manifestation of neurodegenerative diseases or any other neurological disorders that could account for abnormal NfL values.

Group B comprised CSF and blood samples obtained from patients with a confirmed diagnosis of ALSP who were undergoing follow-up with neurologists (PS, PLS, and WBVRP).

Group C consisted of family members of individuals previously diagnosed with ALSP, who had undergone genetic testing confirming the presence of a variant in the *CSF1R* gene. Importantly, these individuals remained asymptomatic throughout the duration of the study.

Group D comprised patients diagnosed with adult-onset leukodystrophy unrelated to the *CSF1R* gene, and who provided consent for participation in the study procedures.

Individuals in Group E were randomly selected from a cohort of 320 patients with sALS undergoing regular follow-up with a neurologist (PS), with an inclusion of up to 15 patients in the study. These patients were actively receiving treatment with Riluzole at the standard dose of 100 mg/day and were not undergoing treatment with Tofersen (Qalsody^®^) or any other investigational product.

All patients gave written informed consent for participation in the study and for this publication, and study procedures were approved by institutional ethics committees (CAAE: 93830518.5.0000.5505).

### 2.2. Biological Samples

Patients in Groups A and C were referred to undergo the collection of a CSF sample, which was obtained on an ice bath, promptly centrifuged, and then stored in 0.5–1 mL aliquots at −80 °C until the time of analysis. To examine biomarkers in patients from Groups B, D, and E, we utilized frozen samples collected during the etiological investigation of these individuals before conducting this study.

### 2.3. Brain MRI

All patients underwent a 3.0 Tesla brain MRI as part of their clinical investigation. This included three-dimensional T1-weighted, diffusion-weighted imaging, gradient-echo T2, T2-weighted imaging (T2-WI), and FLAIR imaging. The primary author reassessed all the images, and a blind neuroradiologist conducted a second evaluation. Due to variations in the timing and imaging acquisition protocols for brain MRI in patients from Groups B, D, and E, no quantitative analysis was conducted. Notably, individuals from Groups A and C underwent brain MRI primarily to rule out the presence of WM abnormalities.

### 2.4. CSF Analysis

The determination of CSF values of chitotriosidase (CHIT) and chitinase 3-like 2 (CHI3L2) were measured as previously described in the literature [17]. The concentrations of NfL in cerebrospinal fluid (CSF) were quantified using commercial enzyme-linked immunosorbent assays (ELISAs from IBL International, Hamburg, Germany). The assays were conducted following the manufacturer’s instructions, exploring a range of 100–10,000 ng/L. Each sample was processed in duplicate, alongside freshly prepared standards, as well as positive and negative controls on each assay plate.

### 2.5. Clinical Data Collection

Participants in Group A underwent independent evaluations by two neurologists (PS and PLS). The assessments included an examination of their past and current health status, neurological exam, and psychiatric evaluation. Additionally, fatigue, depression, anxiety, and their quality of life were also assessed as described for patients from Groups B and D.

The retrospective evaluation of medical records for patients in Groups B, D, and E aimed to comprehend their disease history and medical details. The collected data included information on age at onset, age at diagnosis, time to definitive diagnosis, duration of disease at the time of cerebrospinal fluid (CSF) collection, time to death, leukodystrophy subtype (relevant only to Group D), genetic variants associated with leukodystrophy (for patients in Groups B and D), scores on functional scales such as the Expanded Disability Status Scale (EDSS) [18], Spastic Paraplegia Rating Scale (SPRS) [19], Montreal Cognitive Assessment (MoCA) [20], Amyotrophic Lateral Sclerosis Functional Rating Scale-Revised (ALSFRS-R) (applicable only to Group D) [21], the 8-item Patient Health Questionnaire depression scale (PHQ-8; scale, 0–24) [22], the 7-item Generalized Anxiety Disorder scale (GAD-7; scale, 0–21) [23], Fatigue Severity Scale (FSS) [24], and their health-related quality of life (HRQoL) at the time of CSF collection.

Individuals in Group C were invited to undergo a comprehensive medical interview, including a review of genetic testing that identified a variant in the *CSF1R* gene. The assessment involved questioning about neurological symptoms, neurological examination, and neuropsychiatric evaluation. Fatigue, depression, anxiety, and quality of life assessments were conducted similarly to those in Group A, followed by a subsequent referral for brain MR scanning and CSF collection.

The identification of moderate to severe depression was based on a PHQ-8 cutoff score of ≥10 [22], while mild, moderate, and severe anxiety were identified with GAD-7 scores of 5, 10, and 15, respectively [23].

The evaluation of quality-of-life included patients providing descriptions of their health-related quality of life (HRQoL) using the SF-12 Health Survey Version 2 (SF-12v2). The scale comprises eight sub-domain scores that can be weighted and summarized into two component scores: the physical component summary (PCS) score and the mental component summary (MCS) score. The reference values for PCS and MCS scores for the US population have a mean of 50 and a standard deviation of 10, with a lower score indicating a poorer health status [25,26].

### 2.6. Genetic Tests

All patients in Groups B, C, and D underwent genetic analysis as follow. DNA was extracted from the patients, utilizing peripheral blood leukocytes or saliva. Exome capture was performed using the Agilent Clinical Research Exome v1, following the manufacturer’s instructions. Sequencing procedures were executed on an Illumina NextSeq platform. The obtained exome data were aligned to the GRCh37.75/hg19 reference genome using the Burrows-Wheeler Aligner (BWA; version 0.7.17-r1188). Identification of variants, including single-nucleotide variants (SNVs) and indels, was conducted in accordance with the best practices of the Broad Institute, employing the Genome Analysis ToolKit (GATK, version 3.8-0-ge9d806836) software, and subsequently annotated using Variant Effect Predictor (VEP, version 88.14). All exomes fulfilled the criterion of a minimum of 95% of target bases covered at a depth greater than 10×.

### 2.7. Statistical Analysis

Descriptive statistics were applied, and categorical variables were summarized using counts and percentages of the total population. To assess the normal distribution of continuous variables, the Shapiro–Wilk test was employed. Continuous variables are presented using mean and interquartile range (IQR). Correlation analysis for quantitative and qualitative variables employed Student’s t-test, the chi-squared test, or the Fisher exact test as appropriate. Pearson correlation coefficient was utilized for correlations between continuous variables, where a correlation coefficient of r < 0.3 was considered weak, r = 0.3–0.59 moderate, and r ≥ 0.6 a strong correlation. The statistical analysis was conducted using Stata^®^ 18.0 software, and a two-sided *p*-value < 0.05 was considered statistically significant.

## 3. Results

The study encompassed 63 participants, distributed across various groups: 15 individuals in Group A, designated as the “healthy control”; 11 in Group B referred to as “ALSP group”; 7 in Group C, labeled “asymptomatic *CSF1R* carriers”; 15 in Group D, termed “adult-onset leukodystrophy control group”; and 15 in Group E, denoted “ALS group”. All clinical assessment and biochemical details are exhibited in Table 1 and Table 2.

In cohort A, 12 individuals (80%) identified as Caucasian, 9 (60%) were male, and the mean age at the time of assessment was 33.8 years (26–39.5). The mean scores on the functional scales were 29.4 (29.5–30,0) for the MoCA, 1.03 (0.85–1.2) for the FSS, 7.13 (5.0–9,0) for the PHQ-8, 7.5 (5.5–9.5) for the GAD-7, 52.65 (48.98–55.96) for the PCS, and 49.7 (46.99–52.77) for the MCS component of the SF-12 scale.

Regarding Group B, during the study period, 6 out of the 11 patients (54.5%) were alive. The mean age at the onset of symptoms was 36.7 (31.5–40.5) years, and it took a mean of 13.09 (9.5–15.5) months to establish a definitive diagnosis. Patients in Group B who died had a mean survival time of 57 (45–68) months, while those who were still alive exhibit an average disease duration of 41 (36–44.75) months. At the time of cerebrospinal fluid (CSF) collection, the duration of the disease was 6.9 (5.5–8.5) months. During cerebrospinal fluid (CSF) collection, four patients (36.3%) exhibited an EDSS score ≥ 2.5. The mean scores on the functional scales were 9.5 (6.5–11.5) for the SPRS, 4.17 (3.75–4.60) for the FSS, 21.5 (18.0–25.5) for the MoCA, 11.7 (9.5–14.0) for the PHQ-8, 11.9 (8.5–14.5) for the GAD-7, 36.6 (34.82–41.45) on the PCS, and 30.95 (24.95–34.94) on the MCS of the SF-12 scale.

Within Group C, four individuals (57.1%) were male, with a mean age of 35.7 (33.0–38.5) years. Performance on the functional scales revealed scores of 1.02 (0.9–1.15) on the FSS, 29.4 (29.0–30.0) on the MoCA, 5.2 (4.0–6.5) on the PHQ-8, 6.5 (5.5–7.5) on the GAD-7, 51.2 (46.89–54.54) on the PCS and 48.98 (46.99–48.89) on the MCS of the SF-12 scale.

In group D, 8 out of 15 patients (53.3%) were male, with a mean age at the onset of symptoms of 29.6 (23.0–32.5) years. The mean disease duration at the time of CSF collection was 7.8 (5.0–9.5) months and the mean time for establish a definitive diagnosis was 25.6 (15.5–36.5) months. During CSF collection, the performance on the functional scales showed average values of 11 (6.0–15.0) points on the SPRS, 2.65 (2.05–3.25) on the FSS, 22 (19.5–24.5) on the MoCA, 14.5 (10.5–17.5) on the PHQ-8, 15.2 (12.5–18.0) on the GAD-7, and 35.0 (32.47–38.97) and 33.6 (26.35–39.20) on the PCS and MCS components of the SF12 quality of life scale, respectively. Table 3 summarizes the type of adult-onset leukodystrophy and the associated genetic variants for patients from Group D.

Group E consisted of patients with sALS, who had an average age of 57.8 (54.5–61.5) years at the onset of symptoms. The mean time for CSF collection was 6.2 (4.0–7.5) months and the time for a definitive diagnosis was 7.5 (5.0–9.5) months. At the time of CSF collection, the mean scores were 5.86 (5.65–6.15) on the FSS, 24.86 (23.5–26.5) on the MoCA, 32.33 (30.0–34.5) on the ALSFRS-R, 17.86 (15.0–20.5) on the PHQ-8, 16.4 (14.0–19.0) on the GAD-7, and 27.59 (24.72–28.97) and 27.99 (23.19–31.44) points for the PCS and MCS components of the SF12 scale, respectively.

At the point of cerebrospinal fluid (CSF) collection, there was no statistically significant difference in the age of participants between Groups A and C. However, there was a notable statistically significant difference of 7.12 years [95% CI = 0.53–13.71; *p* = 0.01] in the age at onset of the disease between patients in Groups B and D, with no statistically significant difference observed in the average time for CSF collection. Individuals belonging to Group E exhibited a greater mean age at disease onset compared to those in Group B (mean difference: 21.07; 95% CI = 16.22–25.92, *p* = 0.00001) and Group D (mean difference: 28.2; 95% CI = 22.93–33.46, *p* = 0.00001), respectively.

Concerning clinical functional assessments, no statistically significant differences were observed in any of the assessments when comparing individuals from Group A and Group C. Summaries of the comparisons of mean scores on the assessment scales between Groups B and A, as well as between Groups D and A, are shown in Table 4 and Table 5, respectively.

Patients in Group B exhibited statistically significant difference compared to those in Group D in the FSS (1.51; 95% CI = 0.78–2.25, *p* = 0.0002) and GAD-7 (−3.35; 95% CI = −6.48–−0.22, *p* = 0.01) assessments.

The functional performance of patients in Group B demonstrated a strong positive correlation with the duration of disease up to the time of CSF collection on the SPRS (R = 0.96; *p* = 0.05), PHQ-8 (R = 0.93; *p* = 0.00002), and GAD-7 (R = 0.81; *p* = 0.002) scales. Conversely, there was a strong negative correlation in the MoCA (R = −0.91; *p* = 0.00007), PCS (R = −0.77; *p* = 0.005), and MCS (R = −0.78; *p* = 0.003) assessments.

In comparison to Group E, individuals in Group B exhibited a reduced mean score in the FSS fatigue assessment by −1.68 [95% CI = −2.30–−1.07; *p* = 0.00001], a lower cognitive performance as assessed by MoCA, with a decline of −3.32 [95% CI = −6.61–−0.02; *p* = 0.02], a diminished score in the PHQ-8 by −6.13 [95% CI = −9.52–−2.75; *p* = 0.0006], a decrease in GAD-7 by −4.49 [95% CI = −7.85–−1.12; *p* = 0.005], and an elevated PCS score of 9.01 [95% CI = 4.34–13.68; *p* = 0.0004] on the quality of life scale. Notably, there was no statistically significant difference in the mean score on the MCS of SF-12 quality of life between the two groups.

In relation to CSF biomarkers, individuals in Group B with ALSP showed increased levels of CHIT compared to those in Group A, with a mean difference of 3430.2 [95% CI = 2895.6–3964.8; *p* = 0.00001]. Additionally, they exhibited higher CHI3L2 levels, with a mean difference of 6553 [95% CI = 5152.6–7953.3; *p* = 0.0001], and a more pronounced elevation in NfL levels, indicating a mean difference of 870.5 [95% CI = 545.7–1195.2; *p* = 0.0001].

Individuals in Group C, who were asymptomatic and carry variants in the *CSF1R* gene, show elevated levels of CHIT, with a mean difference of 247.7 [95% CI = 63.4–431.9; *p* = 0.006], and NfL, with a mean difference of 81.3 [95% CI = 3.39–159.3; *p* = 0.02], in comparison to healthy controls in Group A.

Patients with ALSP exhibited higher levels of CHIT, with a mean difference of 3182.5 [95% CI = 2641.8–3723.1; *p* = 0.00001], elevated CHI3L2 levels, showing a mean difference of 6296 [95% CI = 4889.8–7702.2; *p* = 0.00001], and increased neurofilament levels, indicating a mean difference of 789.1 [95% CI = 465.5–1112.7; *p* = 0.0001], compared to asymptomatic carriers in Group C.

When patients with ALSP were compared to those with other adult-onset leukodystrophies, higher levels were observed, with a mean difference of 2215.2 [95% CI = 1327.2–3103.3; *p* = 0.00001] for CHIT, 4858.3 [95% CI = 3192.7–6524.0; *p* = 0.00001] for CHI3L2, and 754.3 [95% CI = 495.2–1013.4; *p* = 0.00001] for NfL in patients with ALSP.

**Figure 1:**
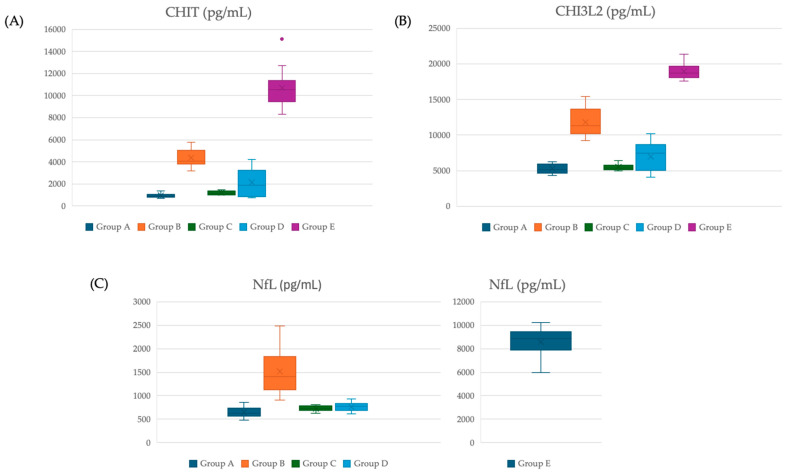
CSF levels of CHIT, CHI3L2 and NfL in each group. Legends: CHI3L2: chitinase 3-like 2; CHIT: chitotriosidase; NfL: neurofilament.

Patients with ALS exhibited statistically significant higher levels of CHIT, CHI3L2, and NfL compared to patients with ALSP. Levels of CSF biomarkers for each group are shown in Figure 1.

In Group B, a strong positive correlation was identified between CHIT levels and CHI3L2 (R = 0.95, *p* = 0.05), as well as with NfL (R = 0.90; *p* = 0001) levels. Additionally, strong correlations were observed between CHIT levels and severity of spasticity measured by the SPRS (R = 0.95; *p* = 0.05), depression levels assessed using PHQ-8 (R = 0.93; *p* = 0.00003), anxiety measured by GAD-7 (R = 0.84; *p* = 0.001), and fatigue evaluated through FSS (R = 0.83; *p* = 0.001). Furthermore, a strong inverse correlation was noted with cognitive function assessed by MoCA (R = −0.91; *p* = 0.0001) and in the PCS (R = −0.74; *p* = 0.008), and MCS (R = −0.71; *p* = 0.01) scores of SF-12. NfL levels exhibited a strong positive correlations with SPRS (R = 0.92; *p* = 0.00005), FSS (R = 0.85; *p* = 0.0009), and PHQ-8 (R = 0.80; *p* = 0.002), and a moderate correlation with GAD-7 (R = 0.65; *p* = 0.02); inverse correlations were observed between NfL levels and cognitive function by MoCA (R = −0.83; *p* = 0.001), and quality of life by PCS (R = −0.88; *p* = 0.0003) and MCS (R = −0.59; *p* = 0.05). In Group D, there was a positive correlation between CHIT and CHI3L2 levels (R = 0.91; *p* = 0.05), while no statistically significant correlation was observed between CHIT and NfL levels or between CHIT levels and performance on functional scales. Notably, NfL levels exhibited a positive correlation solely with the degree of spasticity in the SPRS (R = 0.93; *p* = 0.05).

## 4. Discussion

Chitinases (EC.3.2.1.14) are assigned to families 18 and 19 within the glycosyl hydrolase (GH) superfamily, based on the similarities in their amino acid sequences. GH18 chitinases exhibit widespread expression across archaea, prokaryotes, and eukaryotes. Some members of this family function as active enzymes capable of hydrolyzing chitin and chitodextrin, which are -1,4-linked N-acetyl-D-glucosamine (GlcNAc) oligosaccharides. Chitin, a homopolymer of GlcNAc, stands as the second most abundantly synthesized polymer in nature. It serves as the primary structural component of the extracellular matrix in various organisms, including arthropods, protozoan parasites, nematodes, bacteria, and fungi. Mammals produce authentic chitinases exhibiting enzymatic activity, as well as structurally related chitinase-like proteins (CLPs) that lack enzymatic functionality but possess a high affinity for binding to chitin [27,28,29]. Chitinases in humans exclusively belong to the GH18 family. Among human chitinases are two genuine enzymes, chitotriosidase (CHIT1) and acidic mammalian chitinase (AMCase), along with several chitinase-like proteins (CLPs): chitinase-3-like 1 (CHI3L1), chitinase-3-like 2 (CHI3L2), oviductin-specific glycoprotein (OVGP1), and stabilin-1–interacting CLP (SI-CLP) [27,28,29].

The human chitinase family has undergone extensive scrutiny due to their heightened presence in serum or tissue overexpression during chronic inflammation. Elevated levels of chitinases have been noted in various conditions including infections, chronic inflammation, degenerative disorders, and cancer. Chitinase-like proteins (CLPs) contribute to immunomodulation, thus playing a role in pathological conditions originating from inflammation. Numerous studies have tracked the dynamic changes of chitinases in cerebrospinal fluid, reflecting microglia activation and synaptic and glial dysfunctions, which may be linked to cognitive impairment. CHIT, CHI3L1, and CHI3L2 have received significant attention from both clinical and biological perspectives concerning inflammation-prone brain diseases, and they are now recognized as markers of neuroinflammation across a spectrum of neurodegenerative diseases [27,28,29,30,31,32,33,34,35]. Historically, the CNS was regarded as an immunoprivileged site. However, the current understanding acknowledges that immune responses take place in the brain, engaging both peripheral and local components. This involvement of the immune system is recognized in the pathophysiology of various conditions, predominantly inflammatory diseases like multiple sclerosis, as well as degenerative diseases such as Alzheimer’s disease and amyotrophic lateral sclerosis (ALS) [29,36].

In recent studies, altered levels of CHIT and CHI3L2 have been observed across a spectrum of neurological disorders, including MS, Alzheimer’s disease (AD), ALS, stroke, traumatic brain injury (TBI) and Creutzfeldt–Jakob disease (CJD). These investigations have consistently indicated correlations between these biomarkers and disease activity, progression, and neurological disability. Moreover, they have shown promise in facilitating diagnostic differentiation between affected patients and those with mimicking conditions or healthy individuals [36,37,38,39,40,41,42].

Our findings corroborate recent discoveries, suggesting that CHIT and CHI3L2 serve as reliable biomarkers for distinguishing between healthy controls and patients with ALSP. Furthermore, our evidence suggests that these biomarkers can aid in distinguishing ALSP patients from other adult-onset leukodystrophies, which may present clinical and radiological similarities and thus act as mimickers in clinical practice. Significantly, it is noteworthy that CHIT and CHI3L2 levels exhibited robust statistical correlations with all the clinical assessments conducted in patients with ALSP, suggesting a potential role as prognostic biomarkers.

Furthermore, the difference noted in the levels of CHIT, CHI3L2, and NfL among individuals in Groups B and C suggests that these biomarkers may be valuable in distinguishing symptomatic individuals with ALSP from asymptomatic individuals harboring mutations in the *CSF1R* gene who may exhibit primary neuropsychiatric manifestations or atypical radiological findings unrelated to the ALSP phenotype.

Additionally, our results contribute significant insights into the natural history of ALSP, revealing that these patients experience significant cognitive impairment, disabling fatigue, heightened levels of depression and anxiety, and an overall diminished quality of life compared to normal controls. Notably, individuals with ALSP exhibited higher levels of fatigue and anxiety than those with other adult-onset leukodystrophies. It is also noteworthy that patients with ALSP demonstrated mental health impairment in quality of life comparable to individuals with ALS.

This study revealed that asymptomatic individuals carrying variants in the *CSF1R* gene exhibit elevated levels of CHIT and NfL compared to healthy controls. This elevation suggests signs of axonal damage and activation of an inflammatory response at the molecular level, even in the absence of radiological lesions and clinical manifestations. These findings underscore the necessity for more frequent and long-term evaluations of these patients to detect early signs of the disease and gain insight into the pre-symptomatic phase. The results from Group C highlight the potential of these biomarkers, which could be further explored in larger, prospective studies to enhance our understanding of the pre-symptomatic phase and the mechanisms underlying phenotypic conversion in ALSP. The findings also indicate that NfL, a well-established marker of axonal damage in various neurological diseases [43], serves as a promising biomarker for individuals with ALSP, as one previous study described in the literature [44]. Its elevation compared to normal controls, along with its statistically significant correlation with the disease duration, neurological disability (assessed by SPRS and MoCA), levels of fatigue, anxiety, depression, and impairment in quality of life, parallels observations in other neurological conditions such as ALS and MS [45,46]. This highlights its potential as a responsive biomarker for early therapeutic interventions in clinical trials, similar to its role in ALS [47,48,49].

As documented in the literature, alterations in NfL levels exhibited high sensitivity in detecting axonal damage but lack diagnostic specificity. In conditions where the primary pathology involves glial cells, like microgliopathies, substantial shifts in NfL may not manifest in the early stages of the disease, characterized by minimal axonal dysfunction. Furthermore, NfL levels may not serve as a reliable biomarker to assess the process of microglial activation and function, given its limited expression in microglia [43,50,51].

To better understand the molecular changes in the leukodystrophies group, which are primarily characterized by cellular dysfunction at the level of glial cells, it is essential to assess and characterize biomarkers related to the main pathological process, as exemplified by the analysis of glial fibrillary acidic protein (GFAP) as a biomarker of astrocytic dysfunction in X-linked adrenoleukodystrophy, metachromatic leukodystrophy, and Alexander disease [52,53,54]. Additionally, in the context of ALSP, microglial activity markers such as chitinases may serve as promising biomarkers to explore, as demonstrated by our results and as previously reported by one study in the literature [55].

The study has some limitations, including the small sample size per group, making it challenging to draw inferences for larger populations. In addition, the retrospective and transversal design of the protocol means it is difficult to understand how these biomarkers evolve longitudinally in patients with ALSP and other adult-onset leukodystrophies, thereby significantly restricting the ability to make inferences about the prognostic value of these biomarkers in the studied population. Therefore, conducting additional studies in large international cohorts with diverse clinical presentation profiles would be beneficial to confirm the role of chitinases and neurofilament as biomarkers for ALSP.

## 5. Conclusions

This study represents the first in the literature to establish chitinases (CHIT1 and CHI3L2) as biomarkers for ALSP and to confirm the role of NfL as a good biomarker in the context of primary microgliopathies related to the *CSF1R* gene. Additionally, the findings add a new potential tool to help understand the participation of microglia in leukodystrophies and how we can differentiate the underlying pathological mechanism throughout adult inherited leukoencephalopathies without brain pathology studies.

Our findings further contribute to the understanding of neurofilament as a sensitive biomarker for detecting axonal damage, albeit with limited specificity [50,51]. Moreover, they suggest that ALSP exhibits an underlying progressive inflammatory mechanism that can be tracked through chitinase levels, paralleling observations in more common neurological disorders such as ALS, Alzheimer’s disease, and multiple sclerosis [33,38,39]. These biomarkers may prove beneficial in monitoring clinical responsiveness during the emerging era of therapies involving modulators of the TREM2 pathway to compensate for the *CSF1R* loss of function [15,56].

## Figures and Tables

**Table 1:** Epidemiological and biochemical data.

**Group A (N = 15)**	
Current Age (IQR)	33.8 (26–39.5) years
Gender (M/F)	9 (60%) M/6 (40%) F
CHIT (IQR)	940.8 (794–1027.5) pg/mL
CHI3L2 (IQR)	5258.8 (4723.5–5864.5) pg/mL
NfL (IQR)	644.9 (562.5–718) pg/mL
**Group B (N = 11)**	
Age at Disease Onset (IQR)	36.7 (31.5–40.5) years
Gender (M/F)	7 (63.7%) M/4 (36.3%) F
Death	5 (45.4%)
Duration of disease at CSF collection	6.9 (5.5–8.5) months
CHIT (IQR)	4371 (3882.5–4960) pg/mL
CHI3L2 (IQR)	11,811.9 (10,304.5–13,015) pg/mL
NfL (IQR)	1515.4 (1182.5–1726.5) pg/mL
Genetic Variants	
c.1441C>T (p.Gln481Ter)	N = 3
c.1765G>A (p.Gly589Arg)	N = 1
c.2342C>T (p.Ala781Val)	N = 2
c.2345G>A (p.Arg782His)	N = 2
c.2381T>C (p.Ile794Thr)	N = 1
c.2624T>C (p.Met875Thr)	N = 2
**Group C (N = 7)**	
Current Age (IQR)	35.7 (33–38.5) years
Gender (M/F)	4 (57.1%) M/3 (42.9%) F
CHIT (IQR)	1188.5 (1048.5–1291.5) pg/mL
CHI3L2 (IQR)	5515.8 (5227.5–5709.5) pg/mL
NfL (IQR)	726.2 (700.5–761) pg/mL
**Group D (N = 15)**	
Age at Disease Onset (IQR)	29.6 (23–32.5) years
Gender (M/F)	8 (53.3%) M/7 (46.7%) F
Duration of disease at CSF collection	7.8 (5.0–9.5) months
CHIT (IQR)	2155.8 (1053–3065) pg/mL
CHI3L2 (IQR)	6953.5 (5196–8575) pg/mL
NfL (IQR)	761 (693.5–823.5) pg/mL
**Group E (N = 15)**	
Age at Disease Onset (IQR)	57.8 (54.5–61.5) years
Gender (M/F)	9 (60%) M/6 (40%) F
Duration of disease at CSF collection	6.2 (4.0–7.5) months
CHIT (IQR)	10,698.7 (9660.5–11,244.5) pg/mL
CHI3L2 (IQR)	18,961.8 (18,163–19,449) pg/mL
NfL (IQR)	8570.6 (8142–9347) pg/mL

Legend: CHI3L2: chitinase 3-like 2; CHIT: chitotriosidase; CSF: cerebrospinal fluid; F: female; IQR: interquartile range; M: male; NfL: neurofilament.

**Table 2:** Clinical assessment data.

	Group A	Group B	Group C	Group D	Group E
EDSS	N/A	<2.5: n = 11 ≥2.5: n = 4	N/A	<2.5: n = 5 ≥2.5: n = 10	N/A
FSS (IQR)	1.03 (0.85–1.2)	4.17 (3.75–4.60)	1.02 (0.9–1.15)	2.65 (2.05–3.25)	5.86 (5.65–6.15)
GAD-7 (IQR)	7.5 (5.5–9.5)	11.9 (8.5–14.5)	6.5 (5.5–7.5)	15.2 (12.5–18.0)	16.4 (14.0–19.0)
MoCA (IQR)	29.4 (29.5–30.0)	21.5 (18.0–25.5)	29.4 (29.0–30.0)	22.0 (19.5–24.5)	24.8 (23.5–26.5)
PHQ-8 (IQR)	7.1 (5.0–9.0)	11.72 (9.5–14.0)	5.2 (4.0–6.5)	14.5 (10.5–17.5)	17.8 (15.0–20.5)
SPRS (IQR)	N/A	9.54 (6.5–11.5)	N/A	11.0 (6.0–15.0)	N/A
SF12-PCS (IQR)	52.6 (48.9–55.9)	36.6 (34.8–41.4)	51.2 (46.8–54.5)	35.0 (32.4–38.9)	27.5 (24.7–28.9)
SF12-MCS (IQR)	49.7 (46.9–52.7)	30.9 (24.9–34.9)	48.9 (46.9–48.8)	33.6 (26.3–39.2)	27.9 (23.1–31.4)
ALSFRS-R (IQR)	N/A	N/A	N/A	N/A	32.3 (30.0–34.5)

Legend: ALSFRS-R: Amyotrophic Lateral Sclerosis Functional Rating Scale-Revised; FSS: Fatigue Severity Scale; GAD-7: 7-item Generalized Anxiety Disorder scale; IQR: interquartile range; MCS: mental component summary; MoCA: Montreal Cognitive Assessment; N/A: Not applicable; PCS: physical component summary; PHQ-8: 8-item Patient Health Questionnaire depression scale; SPRS: Spastic Paraplegia Rating Scale; SF-12: 12-item health survey.

**Table 3:** Adult-onset leukodystrophies and genetic variants from Group D.

Patient	Leukodystrophy	Gene	Variant 1	Variant 2
1	X-linked Adrenoleukodystrophy	*ABCD1*	c.311G>A (p.Arg104His)	N/A
2	Metachromatic Leukodystrophy	*ARSA*	c.257G>A (p.Arg86Gln)	c.1283C>T (p.Pro428Leu)
3	Cerebrotendinous Xanthomatosis	*CYP27A1*	c.1183C>T (p.Arg395Cys)	c.1183C>T (p.Arg395Cys)
4	X-linked Adrenoleukodystrophy	*ABCD1*	c.1817C>T (p.Ser606Leu)	N/A
5	Leukoencephalopathy with vanishing white matter	*EIF2B5*	c.338G>A (p.Arg113His)	c.338G>A (p.Arg113His)
6	Leukoencephalopathy with ataxia	*CLCN2*	c.1709G>A (p.Trp570Ter)	c.1709G>A (p.Trp570Ter)
7	Leukoencephalopathy with ataxia	*CLCN2*	c.1709G>A (p.Trp570Ter)	c.1709G>A (p.Trp570Ter)
8	Adult polyglucosan body disease (APBD)	*GBE1*	c.986A>C (p.Tyr329Ser)	c.986A>C (p.Tyr329Ser)
9	Adult polyglucosan body disease (APBD)	*GBE1*	c.986A>C (p.Tyr329Ser)	c.1621A>G (p.Asn541Asp)
10	Alexander Disease	*GFAP*	c.715C>T (p.Arg239Cys)	N/A
11	Cerebrotendinous Xanthomatosis	*CYP27A1*	c.1435C>G (p.Arg479Gly)	c.1435C>G (p.Arg479Gly)
12	Progressive leukodystrophy with ovarian failure	*AARS2*	c.1561C>T (p.Arg521Ter)	c.595C>T (p.Arg199Cys)
13	Progressive leukodystrophy with ovarian failure	*AARS2*	c.1561C>T (p.Arg521Ter)	c.1561C>T (p.Arg521Ter)
14	Cerebrotendinous Xanthomatosis	*CYP27A1*	c.1183C>T (p.Arg395Cys)	c.1028C>G (p.Thr343Arg)
15	Leukoencephalopathy with vanishing white matter	*EIF2B5*	c.271A>G (p.Thr91Ala)	c.271A>G (p.Thr91Ala)

Legend: APBD: Adult polyglucosan body disease.

**Table 4:** Clinical assessment comparison between Groups A and B.

	Group A (Mean)	Group B (Mean)	Mean Difference	*p*-Value
**FSS**	1.03	4.17	−3.13	0.00001
**GAD-7**	7.53	11.90	−4.37	0.005
**MoCA**	29.46	21.54	7.92	0.0001
**PHQ-8**	7.13	11.72	−4.59	0.003
**PCS**	52.65	32.60	16.04	0.00001
**MCS**	49.77	30.95	18.81	0.00001

Legend: FSS: Fatigue Severity Scale; GAD-7: 7-item Generalized Anxiety Disorder scale; MCS: mental component summary of SF-12; MoCA: Montreal Cognitive Assessment; PCS: physical component summary of SF-12; PHQ-8: 8-item Patient Health Questionnaire depression scale.

**Table 5:** Clinical assessment comparison between Groups A and D.

	Group A (Mean)	Group D (Mean)	Mean Difference	*p*-Value
**FSS**	1.03	2.65	−1.62	0.00001
**GAD-7**	7.53	15.26	−7.73	0.00001
**MoCA**	29.46	22.00	7.46	0.00001
**PHQ-8**	7.13	14.53	−7.40	0.00001
**PCS**	52.65	35.00	17.65	0.00001
**MCS**	49.77	33.68	16.09	0.00001

Legend: FSS: Fatigue Severity Scale; GAD-7: 7-item Generalized Anxiety Disorder scale; MCS: mental component summary of SF-12; MoCA: Montreal Cognitive Assessment; PCS: physical component summary of SF-12; PHQ-8: 8-item Patient Health Questionnaire depression scale.

## Data Availability

Data are contained within the article.

## Abbreviations

AD	Alzheimer’s disease
ALS	amyotrophic lateral sclerosis
ALSFRS-R	Amyotrophic Lateral Sclerosis Functional Rating Scale-Revised
ALSP	adult-onset leukoencephalopathy with axonal spheroids and pigmented glia
AMCase	acidic mammalian chitinase
APBD	adult polyglucosan body disease
BWA	Burrows-Wheeler Aligner
CHI3L1	chitinase 3-like 1
CHI3L2	chitinase 3-like 2
CHIT/CHIT1	chitotriosidase
CJD	Creutzfeldt–Jakob disease
CLPs	chitinase-like proteins
CNS	central nervous system
CSF	cerebrospinal fluid
EDSS	Expanded Disability Status Scale
ELISA	enzyme-linked immunosorbent assays
FLAIR	fluid-attenuated inversion recovery
FSS	Fatigue Severity Scale
GAD-7	7-item Generalized Anxiety Disorder scale
GATK	Genome Analysis ToolKit
GD	Gaucher’s disease
GFAP	glial fibrillary acidic protein
GH	glycosyl hydrolase
GlcNAc	N-acetyl-D-glucosamine
HRQol	health-related quality of life
IQR	interquartile range
MCS	mental component summary
MoCA	Montreal Cognitive Assessment
MRI	magnetic resonance imaging
NfL	neurofilament
OPC	oligodendrocyte progenitor cells
OVGP1	oviductin-specific glycoprotein
PCS	physical component summary
PHQ-8	8-item Patient Health Questionnaire depression scale
sALS	sporadic amyotrophic lateral sclerosis
SF-12	12-item Short Form Survey
SF-12v2	SF-12 Health Survey Version 2
SI-CLP	stabilin-1-interacting CLP
SNV	single-nucleotide variant
SPRS	Spastic Paraplegia Rating Scale
T2-WI	T2-weighted imaging
TBI	traumatic brain injury
TREM2	triggering receptor expressed on myeloid cells 2
VEP	variant effect predictor
WM	white matter
